# Physical activity and depression in older adults with and without cognitive impairment

**DOI:** 10.1590/1980-57642018dn12-010002

**Published:** 2018

**Authors:** Kornanong Yuenyongchaiwat, Khajonsak Pongpanit, Somrudee Hanmanop

**Affiliations:** 1 Physiotherapy Department, Faculty of Allied Health Sciences, Thammasat University, Rangsit, Pathumthani, 12120, Thailand

**Keywords:** cognitive impairment, depression, physical activity, older adults, comprometimento cognitive, depressão, atividade física, idosos

## Abstract

Low physical activity and depression may be related to cognitive impairment in the elderly.

**Objetive:**

To determine depression and physical activity (PA) among older adults with and without cognitive impairment.

**Methods:**

156 older adults, both males and females, aged ≥60 years, were asked to complete the Thai Mini-Mental State Examination (Thai-MMSE), a global cognitive impairment screening tool. Seventy-eight older adults with cognitive impairment and 78 older adults without cognitive impairment were then separately administered two questionnaires (i.e., the Thai Geriatric Depression Scale; TGDS and Global Physical Activity Questionnaire; GPAQ). Logistic regression analysis was used to determine the risk of developing cognitive impairment in the groups of older individuals with and without cognitive impairment.

**Results:**

A cross-sectional study of elderly with a mean age of 74.47 ± 8.14 years was conducted. There were significant differences on the depression scale and in PA between older adults with and without cognitive impairment. Further, participants with low PA and high level of depressive symptoms had an increased risk of cognitive impairment (Odds ratio = 4.808 and 3.298, respectively).

**Conclusion:**

Significant differences were noted in PA and on depression scales between older adults with and without cognitive impairment. Therefore, increased PA and decreased depressive symptoms (i.e., having psychological support) are suggested to reduce the risks of cognitive impairment in older adults.

## RESUMO

Baixa atividade física e depressão podem estar relacionados com comprometimento cognitivo em idosos.

**Objetivo:**

Determinar a prevalência de depressão e atividade física (AF) entre idosos com e sem comprometimento cognitivo.

**Métodos:**

156 idosos com idade ≥60 anos completaram o Mini-Exame do Estado Mental tailandês (Thai-MMSE) que é um instrumento de rastreio cognitivo global. Setenta e oito idosos com comprometimento cognitivo e 78 sem comprometimento responderam a dois questionários (versão tailandesa da Escala de Depressão Geriátrica - GSD e do Questionário de Atividade Física Global - GPAQ).

**Resultados:**

houve diferenças na escala de depressão e AF entre os idosos com e sem comprometimento cognitivo. Além disso, participantes com baixa AF e maior frequencia de sintomas depressivos tiveram um aumento no risco de comprometimento cognitivo (OR = 4,808 e 3,298, respectivamente).

**Conclusão:**

Diferenças significativas foram notadas nas escalas de AF e depressão entre idosos com e sem comprometimento cognitivo. O aumento da AF e diminuição de sintomas depressivos (i.e., ter suporte psicológico) sugerem redução no risco de comprometimento cognitivo em adultos idosos.

Currently, the number of older adults in both developed and developing countries is rising, leading to a growing aging society. The World Health Organization (WHO) estimated there are 47 million older individuals with dementia and cognitive impairment globally, more than half (nearly 60%) living in developing countries. The WHO has projected that the number of older adults with cognitive impairment and dementia will be approximately 75 million in 2030 and 132 million by 2050.^1^ The prevalence of age-related health problems is becoming a major problem as the proportion of older adults has markedly increased. It is known that older adults are at significant risk of multiple problems affecting organs, such as cardiovascular, respiratory and memory problems. In addition, there is growing evidence that depression, lack of physical activity and cognitive disorders are common in older adults.^2^^-^ 4 In addition, many studies have found that physical activity (e.g., aerobic exercise) was positively correlated with cognitive function.^5^^-^ 7 In addition, symptoms of depression have also been negatively associated with physical activity level.^8^ Furthermore, a number of studies have revealed that older adults with low physical activity exhibited a high level of depression.^9^^-^ 11 However, little is known regarding the differences in depression and physical activity between older adults with and without cognitive impairment. Therefore, the aim of this study was to explore physical activity and depression scores in older adults with and without cognitive impairment.

## METHODS

### Participants and design

The sample size was calculated from a previous study^12^ using G-power program and statistical power was set at 80%. Therefore, 158 participants were needed to complete the study. Male and female older adults aged ≥60 years were recruited. The Thai Mini-Mental State Examination (Thai-MMSE) was used to classify the participants into two groups (i.e., individuals with and without cognitive impairment); each group comprised 78 older adults. The ethics and protocol were approved by the Ethics committee of Thammasat University, Thailand. All participants agreed to participate by signing the written informed consent form. The study was approved for registration in the Thai Clinical Trials Registry (TCTR) under identification number TCTR20170404001. The cross-sectional study was conducted to determine physical activity level and depression scores in older adults with and without cognitive impairment.

Prior to the study, participants were assessed for cognitive impairment using the Thai-MMSE. The Thai MMSE has been validated against the original version of the MMSE in English by Folstein et al.^13^ The cut-off score on the Thai-MMSE was adjusted based on participants' educational levels. For example, 14 points (out of 23) is the cut-off score for older adults who are illiterate or have not completed elementary school, 17 (out of 30) is the cut-off score for those who have attained complete primary education, and 22 (out of 30) is the cut-off score for those who have completed higher than primary levels of education.^14^ Further, the Thai-MMSE has been widely used to assess cognitive function among the Thai community and is comparable with cognitive impairment screens (e.g., the Mini-Mental State Examination (MMSE) and Clock drawing test (CDT).^15^^-^ 18

The participants with blood pressure (BP) >180/100 mmHg or uncontrolled BP, high heart rate (>120 beats per minute) were excluded. In addition, the participants who had unstable angina, uncontrolled cardiac arrhythmia, chronic cough or had history of cardiovascular disease e.g., stroke or have used cane or walker, were also excluded.

The Thai geriatric depression scale (TGDS) is the standard depression screening tool. The score range for the TGDS is 0-30 points; depression is defined using the cut-off score of 12. The accuracy was 0.94 for females and 0.91 for males.^19^^-^ 21

The Global Physical Activity Questionnaire (GPAQ) was developed by the World Health Organization and has been widely used for detecting physical activity in many countries.^22^ A high level of total physical activity is defined as ≥1,500 MET minutes per week and low physical activity as <600 MET minutes per week.^22^

**Figure 1:**
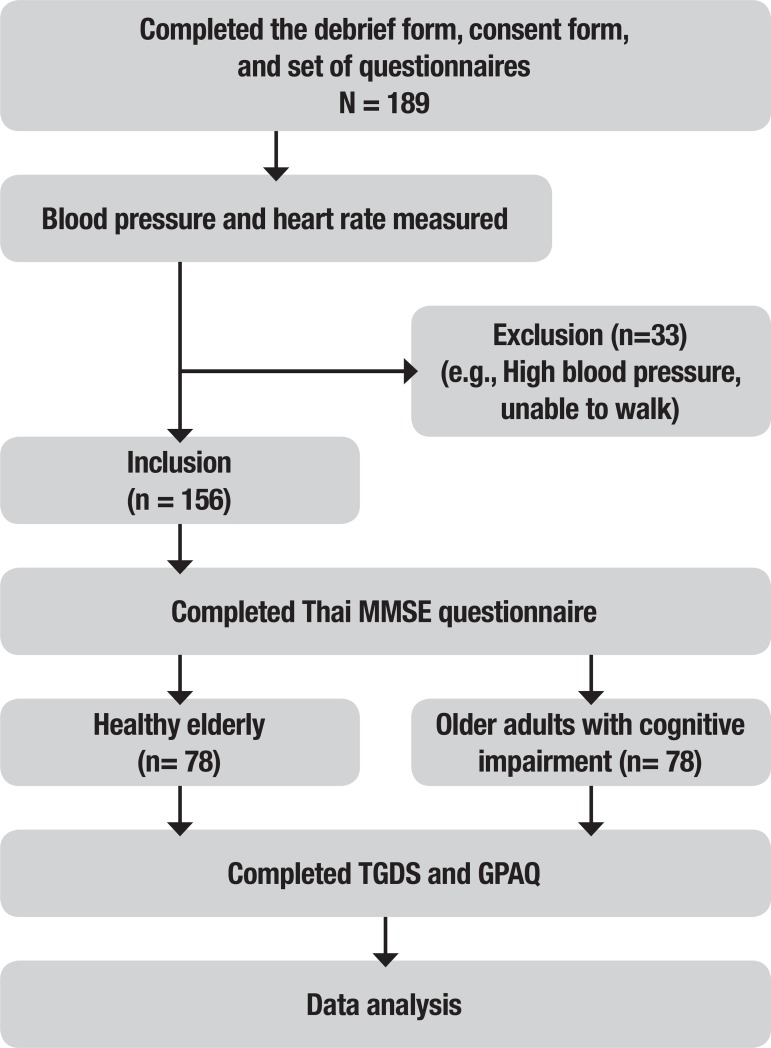
Summary diagram depicting stages of the study. MMSE: Mini-Mental State Examination; TGDS: Thai Geriatric Depression Scale; GPAQ: Global Physical Activity Questionnaire.

Participants were then assessed for the presence of cognitive impairment, depressive symptoms and levels of physical activity by an examiner blinded to the Thai-MMSE, TGDS, and GPAQ, respectively. A summary of the study stages is given in Figure 1.

### Statistical analysis

Descriptive data were presented as mean and standard deviation (SD). Data were verified for normality of distribution (Kolmogorov-Smirnov Goodness-of-Fit test). Independent *t-*tests were performed to examine differences in cardiorespiratory fitness, depression and physical activity scores between older adult with and without cognitive impairment. Chi-square tests were used to compare the number of participants who had symptoms of depression or low physical activity levels with those who had no symptoms or had high/moderate physical activity among the older adults. Further, ANOVA was conducted to determine the contribution of Thai-MMSE score to depression and physical activity. ANCOVA was used to correct for potential risk factors (i.e.; age, gender and educational level). Finally, logistic regression analysis was used to determine independent predictors for cognitive impairment. All analyses were conducted with the SPSS program version 20.0 and the level of statistical significance was considered as *p* <.05.

## RESULTS

### Demographic data in Thai older adults

The mean age of the older adults was 74.47 ± 8.14 years. A total of 156 participants, comprising 83 women and 73 men, completed the TGDS and GPAQ. Regarding educational level, almost half of the participants in this study had completed primary education (43.6%), followed by secondary school (17.3%), no education (14.7%), high school (10.3%) and bachelor or post-graduate level (14.1%). As shown in Table 1, age did not differ significantly between older adults with and without cognitive impairment. However, depression level, as assessed using the TGDS, was significantly higher, and GPAQ scores significantly lower, in older adults with impaired cognitive function compared with older adults with normal cognitive function.

**Table 1:** Comparison between older adults with and without cognitive impairment.

	Cognitive impairment (n = 78)(Mean ± SD)	No cognitive impairment (n = 78)(Mean ± SD)	p-value	Total (n = 156)(Mean ± SD)
Gender (F:M)	39:39	44:34	.261	83:73
Age (year)	75.73 ± 8.61	73.20 ± 7.49	.052	74.47 ± 8.14
Thai-MMSE (scores)	13.38 ± 4.07	25.64 ± 3.87	<.001	19.51 ± 7.31
TGDS (scores)	10.86 ± 5.75	7.33 ± 5.66	<.001	9.10 ± 5.95
GPAQ (MET*min*wk^-1^)	633.59 ± 1205.62	1687.24 ± 2316.00	<.001	1160.42 ± 1914.69

TGDS: Thai geriatric depression scale; GPAQ: global physical activity questionnaire.

### Depression and physical activity in older adults

Analyses were conducted using the depression symptom scores, with a cut-off point of >12 indicating possible depression. Among the participants, 112 (71.79%) older adults were categorized as normal, and 44 (28.21%) were classified as having depressive symptoms. Chi-square tests of significance indicated a statistically significant difference in the proportion of older individuals with and without cognitive impairment for degree of depression (χ^2^ = 10.256, *p* = .001).

In addition, 83 participants (53.21%) were categorized as having low physical activity, 35 (22.44%) moderate, and 38 (24.36%) high physical activity. Chi-square tests of significance indicated that there was a statistically significant difference in the proportion of those with and without cognitive impairment for difference in physical activity levels (χ^2^ = 22.116, *p* <.001).

Table 2 displays the differences in Thai-MMSE scores, physical activity levels, and depressive symptoms between individuals with and without cognitive impairment. ANOVA was conducted to compare Thai-MMSE scores among participants who had depression symptoms. On this analysis, individuals with symptoms of depression had lower Thai-MMSE than those without symptoms of depression; *F* (1,154) = 12.186, *p* = .001, ηp*^2^* = .073. ANCOVA revealed that these relationships remained after adjusting for age, gender and educational levels; individuals with symptoms of depression still had lower Thai-MMSE than individuals without symptoms of depression; *F* (1, 151) = 7.310, *p* = .008, ηp*^2^* = .046.

**Table 2:** Thai-MMSE scores according to depression and physical activity.

Variables	Thai-MMSE	F (test)	hp^2^	F (test) after adjustment^#^	hp^2^ after adjustment^#^
TGDS		12.186**	.073	7.310**	.047
Depression (n = 44)	16.36 ± 6.76				
No depression (n = 112)	20.75 ± 7.18				
PA		12.085***	.136	12.747***	.145
Low (n = 83)	17.00 ± 6.88				
Moderate (n = 35)	22.77 ± 7.03				
High (n = 38)	22.00 ± 6.57				

Thai-MMSE: Thai mini-mental state examination. TGDS: Thai geriatric depression scale. PA: physical activity.

# After adjustment for age, gender and educational level

** *p* <.01;

*** *p* <.001.

ANOVA was conducted to compare Thai-MMSE scores between participants who had different GPAQ. Individual with low physical activity had lower Thai-MMSE scores than those with moderate-to-high physical activity; *F* (2,153) = 12.085, *p* <.001, ηp*^2^* = .136. ANCOVA was conducted to compare the difference in the three physical activity levels whilst controlling for age, gender and educational level; there was a significant difference in Thai-MMSE scores [F(2,150) = 12.747, *p* <.001, ηp*^2^* = .145] between the levels of physical activity. Post-hoc tests showed a significant difference between low and moderate (*p* <.001) and low and high physical activity (*p* <.001) levels. In conclusion, physical activity was associated with Thai-MMSE scores in the Thai participants even after controlling for age, sex and educational level.

In summary, both depression symptoms and levels of physical activity levels were associated with Thai-MMSE in the Thai participants even after controlling for age, sex and educational level.

To determine the risk of developing cognitive impairment, a logistic regression analysis was performed. Age, gender, levels of physical activity (i.e., low physical activity, and moderate-to-high physical activity) and depression symptoms were evaluated (Table 3). Low physical activity was found to be a major risk factor (odds ratio (OR) 4.808, confident interval (CI) 2.439-9.479, *p* <.001) while female gender was not an independent risk factor for cognitive impairment (OR = .773, CI = .411-1.451, *p* = .423). Further, Individuals with depression were at higher risk for developing cognitive impairment (OR = 3.298, CI = 1.560-6.971, *p* = .001). In addition, participants in the ≥80 years age group had an OR of 5.562 (CI = 1.187-6.389, *p* = .018) for cognitive impairment compared to those in the 60-69 years age group.

**Table 3:** Results of logistic regression analysis for risk factors of cognitive impairment.

Risk factors for cognitive impairment	Odds ratio	95% CI for OR	p-value
Gender	Reference group - male		
• Female	.773	.411 to 1.451	.423
Age groups (years)	Reference age group 60-69 years		
• 70-79	1.137	.527 to 2.453	.744
• ≥80	5.562	1.187 to 6.389	.018
GPAQ	Reference group - moderate-to-high PA		
• Low PA	4.808	2.439 to 9.479	<.001
TGDS	Reference group - no depression		
• Depression	3.298	1.560 to 6.971	.001

CI: confidence interval; OR: odds ratio; PA: physical activity; GPAQ: Global Physical Activity Questionnaire; TGDS: Thai Geriatric Depression Scale.

## DISCUSSION

The present study determined the physical activity level and depressive symptoms in older adults with cognitive and without cognitive impairment. The present study indicated that older adults with cognitive impairment are less active and have more depressive symptoms compared to those without cognitive impairment.

Results of the present study are consistent with previous studies. Several studies have suggested that physical activity can prevent or counteract cognitive decline.^5^^-^ 6 The meta-analysis of 18 randomized controlled trials reported that improved cognitive function in patients with dementia was associated with increased physical activity such as aerobic exercise.^7^ Several prospective cohort studies have reported that older adults with higher level of physical activity had slower decline in cognitive function over both short-term (i.e., 2.5 years)^23^ and long-term (i.e., over 6 years)^24^ follow-ups compared with individuals with lower levels of physical activity. Bherer, Erickson, & Liu-Ambrose^5^ (2013) concluded that physical activity is associated with brain structures (e.g., induced angiogenesis, neurogenesis, synaptogenesis) and impacts functional ability and cognitive performance (executive functions, processing speed, and working memory) in older adults. Tan et al.^25^ (2017) examined the relationship between physical activity and brain volume and found that levels of physical activity and total cerebral and hippocampal brain volume were related; where increased aged was associated with decreased volume of hippocampus and cerebral structures. Further, they suggested that decreases in total brain and hippocampal volumes could be prevented by a higher level of physical activity. This may, in part, explain why physical inactivity was observed in older adults with cognitive impairment in the present study. However, the potential dose response of intensity and duration of physical activity levels to maintain brain morphology is unclear.^5^^,^ 25

Reports in older adults have shown a relationship between greater depressive symptoms and poor physical and social functions. Several studies have found that individuals with poor cognitive functioning exhibit mental health problems, including depression.^26^^-^ 28 Furthermore, Barnes et al.^27^ (2006) found that depressive symptoms were associated with increased risk of mild cognitive impairment after 6 years of follow-up in a population-based survey; participants with higher depression scores had a high risk for mild cognitive impairment. Yaffe et al.^28^ (2001) reported that older women with greater physical activity (i.e., more walking) experienced less cognitive decline during the 6 to 8 year follow-up. In addition, after controlling for age, educational level, health status, smoking and hormonal use (i.e., estrogen), women with higher physical activity remained at lower risk for decreased cognitive function. Wassink-Vossen et al.^29^ (2014) reported that older adults with depression symptoms had lower physical activity compared to non-depressed older adults. In addition, they found that physical inactivity in elderly with depressive symptoms displayed more functional limitations. Penninx et al.^30^ (1999) compared older adults with and without depressive symptoms (using the Center for Epidemiological Studies Depression Scale) and found that depressed older adults had lower physical activity compared to non-depressed elderly. Further, an association between lower levels of physical activity and functional limitations was identified, resulting in a risk of physical disability in long-term follow-up. van Rossum & Koek^31^ (2016) reported that low physical activity was associated with functional disability in cognitive impairment. Moreover, there is a body of evidence supporting that increased physical activity is associated with decreased depressive symptoms and mental health problems.^8^^,^ 32 ^-^ 34

Several lines of evidence have demonstrated that modifiable risk factors for cognitive decline include depression, low educational level and physical inactivity.^35^^-^ 40 However, evidence exists with long follow-up showing no difference in physical activity levels between individuals with and without dementia between 28 and 10 years before diagnosis.^41^ The authors revealed that a marked reduction in physical activity of individuals with dementia began nine years before diagnosis. Further, levels of physical activity rapidly declined in the years leading up to diagnosis of dementia.^41^ Therefore, they suggested that changes in physical activity level should be recognized as one of the preclinical manifestations of dementia symptoms.^41^ In addition, the risk of developing cognitive impairment can be reduced by sufficient physical activity or exercise. Therefore, it is important to focus on these risk factors. As this study has confirmed, lack of exercise or physical activity is a major risk factor. Older adults who had low physical activity had a higher prevalence of cognitive impairment.

The study adds to a growing body of evidence that support the relationship between physical activity and mental health (i.e., depression) in older adults with and without cognitive impairment. Further, the present study suggests that individuals who exhibited decline in cognition had low physical activity and higher depression scores; therefore; the cognitive function in elderly persons might be enhanced by improving physical activity and this may decrease depression in older adults. For example, 150 minutes of moderate-intensity physical activity per week, or 50 minutes for 3 days per week, for 6 months (e.g., walking, light strength training exercise) improved cognitive function in older adults.^42^

Some limitations of the present study should be noted. First, this study is based on a cross-sectional study design, precluding determination of cause and effect between predictors of interests (i.e., physical activity and depression) and an outcome (i.e., cognitive function). Prospective cohort studies with longitudinal follow-up can determine the relationships between physical activity and depression and cognitive impairment. The study asked participants to self-rate physical activity and depression using a questionnaire, providing only a subjective ratings of depression levels and physical activity levels during the previous week. Therefore, the questionnaires also warrant further study regarding their recall bias, particularly in older age groups. Further, studies examining physical activity and depression using objective methods instead of subjective measures are needed. Differences in age, gender and educational levels may, in part, account for physical activity, depression and cognitive impairment. However, these relationships were adjusted for physical activity, depression and educational level.

In summary, older adults with cognitive impairment had higher depression scores and lower physical activity than older adults without cognitive impairment. Therefore, increased physical activity and lower depression could potentially have a beneficial effect on cognitive function in older adults.

## References

[1] WHO (2017). Dementia: Fact Sheet No. 362.

[2] Buchtemann D, Luppa M, Bramesfeld A, Riedel-Heller S (2012). Incidence of late-life depression: a systematic review. J Affect Disord.

[3] Diniz BS, Butters MA, Albert SM, Dew A, Reynolds CF (2013). Late-life depression and risk of vascular dementia and Alzheimer's disease: systematic review and meta-analysis of community-based cohort studies. Br J Psychiatry.

[4] Murtagh EM, Murphy MH, Murphy NM, Woods C, Nevill AM, Lane A (2015). Prevalence and correlates of physical inactivity in community-dwelling older adults in Ireland. PLos One.

[5] Bherer L, Erickson KI, Liu-Ambrose T (2013). A review of the effects of physical activity and exercise on cognitive and brain functions in older adults. J Aging Res.

[6] Larson EB, Wang L, Bowen JD, McCormick WC, Teri L, Crane P (2006). Exercise is associated with reduced risk for incident dementia among persons 65 years of age and older. Ann Intern Med.

[7] Groot C, Hooghiemstra AM, Raijmakers PG, van Verckel BN, Scheltens P (2015). The effect of physical activity on cognitive function in patients with dementia: A meta-analysis of randomized control trials. Ageing Res Rev.

[8] Yuenyongchaiwat K (2016). Effects of 10,000 daily steps a day on physical and mental health states in overweight participants with sedentary lifestyle in community dwelling: A preliminary study. Braz J Phys Ther.

[9] Davidson RJ, Lewis DA, Alloy LB, Amaral DG, Bush G, Cohen JD (2002). Neural and behavioral substrates of mood and mood regulation. Biol Psychiatry.

[10] Barnes DE, Alexopoulos GS, Lopez OL, Williamson JD, Yaffe K (2006). Depressive symptoms, vascular disease, and mild cognitive impairment. Arch Gen Psychiatry.

[11] Yaffe K, Barnes D, Nevitt M, Lui LY, Covinsky (2001). A prospective study of physical activity and cognitive decline in elderly women: women who walk. Arch Intern Med.

[12] Boyle PA, Buchman AS, Wilson RS, Leurgans SE, Bennett DA (2009). Association of muscle strength with the risk of Alzheimer disease and the rate of cognitive decline in community-dwelling older persons. Arch Neurol.

[13] Folstein MF, Folstein SE, McHugh PR (1975). "Mini-mental state". A practical method for grading the cognitive state of patients for the clinician. J Psychiatr Res.

[14] Thai Cognitive Test Development Committee 1999 (2002). Mini-Mental State Examination-Thai 2002.

[15] Kuha O (2008). The comparison of relation of Mini Mental Status Examination Thai version (MMSE-Thai) 2002 and Thai Mini-Mental State Examination; TMSE in dementia screening.

[16] Silpakit O, Silpakit C, Pukdeenaul P (2007). A comparison study of cognitive impairment screening tools: CDT, IQCODE vs MMSE. Siriraj Med J.

[17] Wongpakaran N, Wongpakaran T (2013). Cornell Scale for depression in dementia: Study of residents in a northern Thai long-term care home. Psychiatry Investig.

[18] Wongpakaran N, Wongpakaran T, Bookamana P, Pinyopornpanish M, Maneeton B, Lerttrakarnnon P (2011). Diagnosing delirium in elderly Thai patients: Utilization of the CAM algorithm. BMI Family Practice.

[19] Train the Brain forum Committee (1994). Thai Geriatric Depression Scale-TGDS. Siriraj Hospital Gazette.

[20] Muangpaisan W, Assantachai P, Intalapaporn S, Pisansalakij D (2008). Quality of life of the community-based patients with mild cognitive impairment. Geriatr Gerontol Int.

[21] Muangpaisan W, Intalapaporn S, Assantachai P (2008). Neuropsychiatric symptoms in the community-based patients with mild cognitive impairment and the influence of demographic factors. Int J Geriatr Psychiatry.

[22] Armstrong T, Bull F (2006). Development of the World Health Organization Global Physical Activity Questionnaire (GPAQ). J Public Health.

[23] Aichberger MC, Busch MA, Reischies FM, Ströhle A, Heinz A, Rapp MA (2010). Effect of physical inactivity on cognitive performance after 2.5 years of follow-up: longitudinal results from the survey of health, ageing, and retirement (SHARE). GeroPsych.

[24] Barnes DE, Yaffe K, Satariano WA, Tager IB (2003). A longitudinal study of cardiorespiratory fitness and cognitive function in healthy older adults. J Am Geriatr Soc.

[25] Tan ZS, Spartano NL, Beiser AS, DeCarli C, Auebach SH, Vasan RS (2017). Physical Activity, Brain Volume, and Dementia Risk: The Framingham Study. J Gerontol A Biol Sci Med Sci.

[26] Davidson RJ, Lewis DA, Alloy LB, Amaral DG, Bush G, Cohen JD (2002). Neural and behavioral substrates of mood and mood regulation. Biol Psychiatry.

[27] Barnes DE, Alexopoulos GS, Lopez OL, Williamson JD, Yaffe K (2006). Depressive symptoms, vascular disease, and mild cognitive impairment. Arch Gen Psychiatry.

[28] Yaffe K Barnes D, Nevitt M Lui LY, Covinsky (2001). A prospective study of physical activity and cognitive decline in elderly women: women who walk. Arch Intern Med.

[29] Wassink-Vossen S, Collard RM, Oude Voshaar RC, Comijs HC, de Vocht HM, Naarding P (2014). Physical (in)activity and depression in older people. J Affect Disord.

[30] Penninx BW, Leveille S, Ferrucci L, van Eijk JT, Guralnik JM (1999). Exploring the effect of depression on physical disability: longitudinal evidence. Am J Public Health.

[31] van Rossum ME, Koek HL (2016). Predictors of functional disability in mild cognitive impairment and dementia. Maturitas.

[32] Deslandes AC, Moraes H, Alves H, Pompeu FA, Silveira H, Mouta R (2010). Effect of aerobic training on EEG alpha asymmetry and depressive symptoms in the elderly: a 1-year follow-up study. Braz J Med Biol Res.

[33] Deslandes A, Moraes H, Ferreira C, Veiga H, Silveira H, Mouta R (2009). Exercise and mental health: many reasons to move. Neuropsychobiology.

[34] Flicker L, Lautenschlager NT, Almeida OP (2006). Healthy mental ageing. J Br Menopause Soc.

[35] Barnes DE (2011). The projected impact of risk factor reduction on Alzheimer's disease prevalence. Lancet Neurol.

[36] Pellegrino LD, Peters ME, Lyketsos CG, Marano CM (2013). Depression in Cognitive Impairment. Curr Psychiatry Rep.

[37] Baumgart M, Synder HM, Carrillo MC, Fazio S, Kim H, Johns H (2015). Summary of the evidence on modificable risk factors for cognitive decline and dementia: A population-based perspective. Alzheimers Dement.

[38] Blondell SJ, Hammersley-Mather R, Veerman JL (2014). Does physical activity prevent cognitive decline and dementia? A systematic review and metaanalysis of longitudinal studies. BMC Public Health.

[39] Xu W, Tan L, Wang HF, Jiang T, Tan MS, Tan L (2015). Meta-analysis of modifiable risk factors for Alzheimer's disease. J Neurol Neurosurg Psychiatry.

[40] Norton S, Matthews FE, Barnes DE, Yaffe K, Brayne C (2014). Potential for primary prevention of Alzheimer's disease: an analysis of populationbased data. Lancet Neurol.

[41] Sabia S, Dugravot A, Dartigues JF, Abell J, Elbaz A, Kivimaki M, Singh-Manoux A (2017). Physical activity, cognitive decline, and risk of dementia: 28 year follow-up of Whitehall II cohort study. BMJ.

[42] Lautenschlager NT, Cox KL, Flicker L, Foster JK, van Bockxmeer FM, Xiao J (2008). Effect of physical activity on cognitive function in older adults at risk for Alzheimer disease: A randomized trial. JAMA.

